# Impact of Dry Eye Disease on the Uncorrected Distance Visual Acuity after Small Incision Lenticule Extraction

**DOI:** 10.3390/jcm12196179

**Published:** 2023-09-25

**Authors:** Yan Shen, Jiajia Wang, Xingtao Zhou, Zhiqiang Yu, Jiaxu Hong, Qihua Le

**Affiliations:** 1 Department of Ophthalmology, Eye, Ear, Nose & Throat Hospital of Fudan University, Shanghai 200031, China; shenyan74432@163.com (Y.S.); wangjiajia0119@163.com (J.W.); doctzhouxingtao@163.com (X.Z.); zhiqiang.yu@fdeent.org (Z.Y.); 2 Research Center, Eye, Ear, Nose & Throat Hospital of Fudan University, Shanghai 200031, China; 3 Myopia Key Laboratory of Ministry of Health, Eye, Ear, Nose & Throat Hospital of Fudan University, Shanghai 200031, China

**Keywords:** dry eye disease, small-incision lenticule extraction, visual acuity, refractive status

## Abstract

The aim of this study was to explore the impact of dry eye disease (DED) on the uncorrected distance visual acuity (UDVA) and refractive status after small incision lenticule extraction (SMILE). This prospective cohort study enrolled 29 patients (DED group, 11 eyes; non-DED group, 18 eyes) who underwent SMILE in our center from July to September 2022. The examinations on DED, refractive status and UDVA were performed before surgery, and on day 7 and 20 after surgery. The results showed that on day 20 after SMILE, subjects in the non-DED group reported greater changes of ocular surface disease index value increase and tear-film breakup time reduction compared to baseline than those in the DED group (*p* < 0.001 and *p* = 0.048, respectively). Compared to preoperative status, DED patients had greater improvements of UDVA and better optometric outcomes on day 20 after surgery than non-DED subjects (*p* = 0.008 and 0.026, respectively). Multiple linear regression analysis showed age, contact lens daily wearing time, and tear meniscus height before surgery were of the highest value to predict UDVA on day 20 after SMILE in contact lens wearers (*p* = 0.006, 0.010 and 0.043, respectively). In conclusion, preoperative tear function could affect UDVA after SMILE. The impact of DED on UDVA and refraction should be taken into consideration before surgery.

## 1. Introduction

Myopia is one of the most common ocular diseases in Eastern Asians, especially adolescents and young people [1,2]. More than 6 million people have been reported to undergo small incision lenticule extraction (SMILE) globally since 2011, among which more than half were performed in China [3]. Several factors have been shown to potentially affect visual function postoperatively, including surgery types [4,5], higher-order aberration [4,6], pupil size [7], and dry eye disease (DED) [8,9].

DED is a multifactorial disease of the ocular surface characterized by a loss of homeostasis of the tear film, and accompanied by ocular symptoms, in which tear film instability and hyperosmolarity, ocular surface inflammation and damage, and neurosensory abnormalities play etiological roles [10]. The main clinical manifestations of DED are ocular discomfort, dryness, burning sensation, grittiness, photophobia, pain, visual disturbance due to partial or total tear film instability, increased tear film osmolarity, and subacute inflammation of the ocular surface [11]. As one of the most common ocular surface diseases impairing vision-related quality of life, its prevalence varies from 4.29% to 50.33% in the Chinese population [12]. Many risk factors have been confirmed to be closely related to the development and deterioration of DED, among which refractive surgery is an important one, especially in Asians [13].

DED and corneal refractive surgery influence each other. On one hand, due to a strong willingness to free themselves from glasses, most patients used to be contact-lens wearers, which is an already-known risk factor of DED [13]. Therefore, a great number of patients have had DED before corneal refractive surgery. On the other hand, DED is one of the most common complications after corneal refractive surgery, which might be attributed to many factors including impaired corneal sensation and decreased nutrition on corneal epithelium due to the damage of the sub-basal and stromal nerve plexus, decreased mucin secretion due to the damage of goblet cells, and abnormal aqueous and lipid tear production due to reduced blinking frequency. All of them affect the quality and quantity of tear films [14,15,16,17]. Compared with SMILE, laser-assisted in situ keratomileusis (LASIK), which is involved with flap creation and photoablation of corneal stroma, induces more keratocyte apoptosis and inflammation, and higher risks of flap-related complications [18,19]. Reinstein DZ et al. confirmed that after the surgery, SMILE could maintain stromal tensile strength better than LASIK [20]. Nevertheless, the reports on DED after SMILE are not rarely seen even though it has a less negative impact on the corneal sub-basal nerve plexus, biomechanical stability and ocular surface microenvironment [17,18,21,22,23,24,25,26]. Moreover, the impact of preoperative DED on the prediction of uncorrected distance visual acuity (UDVA) and refractive status after SMILE has not been fully addressed up till now.

Therefore, we performed this prospective study in order to investigate the impact of pre-operative DED on postoperative UDVA and refractive status, and explore the predictive potential of DED parameters on UDVA.

## 2. Materials and Methods

### 2.1. Study Population

This prospective cohort study was approved by the Institutional Review Board of Eye, Ear, Nose & Throat Hospital of Fudan University and conformed to the tenets of the Helsinki Declaration. A total of 29 subjects who underwent SMILE in Eye, Ear, Nose & Throat Hospital of Fudan University from July to September 2022 were enrolled. Written informed consents were obtained from all participants. Eleven patients met the following criteria of DED as previously described [27]: (1) a frequent or sustained occurrence of at least two dry eye symptoms (ocular discomfort/foreign body sensation, photophobia, grittiness, pain, dryness, blurred and fluctuating vision); (2) the presence of any two of the following three signs: (i) tear film breakup time <10 s, (ii) Schirmer I test (SIT) <10 mm/5 min, and (iii) corneal fluorescein staining (CFS) score ≥ 1 [28,29]. They were assigned in a preoperative DED group (*n* = 11), and the other 18 patients were in a non-DED group (*n* = 18).

The exclusion criteria included: (1) <18-year-old; (2) pregnancy or in lactation; (3) history of autoimmune diseases or ocular trauma; (4) active ocular or periocular infection or inflammation; (5) concomitant lid margin abnormality; (6) history of ocular or periocular surgery within 6 months before enrollment; (7) history of artificial tear usage within 2 weeks before enrollment; (8) history of lacrimal punctal occlusion; (9) patients who could not cooperate with the examinations and surgery.

### 2.2. Ocular Examinations

#### 2.2.1. Order of Ocular Examinations

Eligible subjects were required to fulfil a self-reported questionnaire, Ocular Surface Disease Index (OSDI), before ocular examination to avoid the influence of clinical examinations on their responses. Sociodemographic data were also obtained prior to ocular examination, which included age, gender, occupation and history of contact lens wear. Then, all participants underwent a thorough ocular examination including best corrected visual acuity (BCVA), slit-lamp biomicroscopy, direct ophthalmoscopy, fundus photography, tonometry, optometric examination, and Oculus Keratograph 5M (Wetzlar, Germany). Fluorescence tear film breakup time (FBUT) and CFS were performed during slit-lamp biomicroscopy examination. SIT without anesthesia was finally performed when all the other examinations were finished so as to avoid the impact on corneal epithelium and fluorescence staining. These examinations were performed before surgery, and repeated on day 7 and 20 after surgery. Moreover, UDVA and BCVA were obtained after the surgery, both of which were evaluated with Standard for logarithmic visual acuity charts (GB/T 11533-2011, China) [30]. The eye with DED was included in patients with monocular DED, while the more severe eye was enrolled if the patients were bilaterally affected. The primary endpoints were UDVA and refractive status on Day 20 after SMILE. DED parameters on Day 7 and Day 20 postoperatively were also evaluated.

#### 2.2.2. OSDI

OSDI questionnaire consists of three sections (a total of 12 questions), and evaluates the severity of ocular discomfort symptoms, visual functions related life quality and environmental triggers in the recent week. OSDI scores = (sum of scores for questions answered × 25)/(number of answered questions) [31].

#### 2.2.3. Oculus Keratograph 5M

Oculus K5M was used to obtain non-invasive breakup time (NIBUT), tear meniscus height (TMH), lipid layer color (LLC), lipid layer uniformity (LLU), and meibomian gland (MG) loss. After blinking twice in a dark room, the patients were required to keep their eyes open as long as they could until the next blink took place. Then, the upper and lower eyelids were everted by the same operator to capture the meibography with infrared system. The examinations were repeated three times.

#### 2.2.4. Slit-Lamp Biomicroscopy

All subjects underwent a slit-lamp examination. Those having any other ocular abnormalities that might potentially interfere with the tear film were excluded. FBUT and CFS were assessed under cobalt blue light during slit-lamp examination as previously reported [27].

#### 2.2.5. SIT

Without topical anesthesia, Schirmer paper strips (5 × 40 mm, Jingming, Tianjin, China) were folded at the notch and placed in the 1/3 of the external lower conjunctival sac cautiously. Then, the patients were asked to close their eyes gently for 5 min. The length of wetting by tears from the notch was measured and recorded.

### 2.3. Image Analysis and Measurement

#### 2.3.1. Assessment of DED Parameters Obtained by K5M

DED parameters were measured based on the placido rings projected on the tear film. The changes of tear film during two blinks were presented as color-coded tear maps, in which the color closest to red indicated the location with the most instable tear film. The duration between the first blink and tear film break-up at this location was recorded as the first NIBUT. The mean value of the first NIBUT in different zones of the cornea was calculated as the average NIBUT. TMH was measured from the lower lid margin with the application of a caliper tool in the customized software, just as previously described [32]. The measurement of NIBUT and TMH was performed on three scans and the average values were calculated.

Based on the image captured immediately after blinking, LLC and LLU were evaluated. LLC was classified into five colors: multicolor, red-green, both of which were considered as normal, and blue-grey, hoary, and achromatic, which were abnormal. LLU was divided into even distribution and uneven distribution, which were assigned the scores 0 and 1, respectively. The grades of MG loss were scored according to the proportion of MG dropout area over the entire gland area. Score 0, 1, 2, and 3 represented no dropout, dropout area ≤ 1/3, 1/3 < dropout area ≤ 2/3, and dropout area > 2/3, respectively [33].

#### 2.3.2. Measurement of FBUT

The participants were asked to naturally blink several times until the cornea was fully covered with fluorescein sodium solution. Then, they were told to keep eyes open as long as possible under the cobalt blue light. The duration from the last blink to the first black spot on the corneal surface was measured and recorded as FBUT. The outcome of FBUT was the average of three repeated examinations for each eye.

#### 2.3.3. Evaluation of CFS

CFS was assessed within 1–3 min after fluorescein instillation [34]. Based on the National Eye Institute grid, the cornea was divided into 5 quadrants (central, superior, inferior, nasal and temporal) and corneal epithelial staining in each quadrant was evaluated and scored 0–3 according to the following criteria: 0, no staining; 1, <15 dots; 2, 16–30 dots; 3, >30 dots or strip/bulk staining or corneal filaments [28]. The total score ranged from 0–15, and the score ≥ 1 was considered as positive.

### 2.4. Surgical Procedure and Postoperative Treatment

The procedure of SMILE was performed as previously described [35,36]. In brief, 500-kHz femtosecond laser system (Visumax; Carl Zeiss, Jena, Germany) was set to 110–160 μm as the intended cap thickness and 7.6 mm as the intended cap diameter. The lenticule optical zone diameter was set between 6.1 and 6.8 mm based on the diameter of scotopic pupil. With a 2 mm wide small incision, the vertical side cut was created at 90- or 120-degree superior cornea. The refractive stromal lenticule was dissected after laser cutting and extracted through the incision. After the surgery, all patients were administered 0.5% levofloxacin eye drops 4 times/d for 7 days, and 0.1% fluorometholone eye drops tapered every 3 days from 8 times/d to once a day within 3 weeks.

### 2.5. Statistical Analysis

Stata 16.0 software (Stata Corp., College Station, TX, USA) and SPSS Statistics 22.0 (IBM Corp., Armonk, NY, USA) was used for data analysis. The Shapiro–Wilk test was used to test the normality of data. The data with normal distribution were presented as means ± standard deviation, otherwise as median (range). Student’s t or *t*’ test, Wilcoxon rank sum test, and Fisher’s exact method were used to analyze normally distributed data, non-normally distributed data and enumeration data, respectively. As for the comparison on data in one group among different time points, two-way analysis of variance, Friedman test and Cochran’s Q test were performed. Bonferroni correction was used in subgroup pairwise comparisons. Regression analysis was performed in contact lens wearers. A total of 14 variables were included in the univariate linear regression analysis, including age, wearing duration, wearing frequency, daily wearing time, UDVA, spherical equivalent (SE), OSDI value, SIT, FBUT, CFS score, first NIBUT, average NIBUT, TMH, and grade score of MG loss. Variables with *p* values less than 0.35 in univariate linear regression analysis were selected for multiple linear regression analysis to explore the predictive parameters for UDVA. A *p* value < 0.05 was considered statistically significant.

## 3. Results

A total of 29 patients (12 males and 17 females, 29 eyes) with a median age of 24 (range: 17–39) years were enrolled. Among them, 15 eyes had the history of wearing contact lens, and the majority was soft contact lens (14/15, 93.33%). Preoperative LogMAR UDVA of all subjects was 1.06 ± 0.31, and SE was −5.08 ± 1.64 diopters (D). The demographic data and clinical characteristics of the DED group and non-DED group were summarized in Table 1. Before the surgery, subjects in the DED group reported a worse UDVA, a higher OSDI score, a lower SIT value and a higher proportion of abnormal LLC than those in the non-DED group (*p* = 0.036, <0.001, 0.041 and 0.008, respectively).

### 3.1. Postoperative UDVA and Refractive Status

On day 20 after surgery, the LogMAR UDVA and SE of all subjects were −0.03 ± 0.08 and (−0.22 ± 0.39) D, both of which were significantly improved compared to the baseline values (both *p* < 0.001). However, the comparisons on UDVA between the DED group and non-DED group did not have significant differences (*p* = 0.248). It was unexpected that, compared to preoperative status, DED patients had greater improvements of UDVA and better optometric outcomes on day 20 after SMILE than non-DED subjects (*p* = 0.008 and 0.026, respectively) (Table 2).

### 3.2. Postoperative DED Parameters

**Figure 1:**
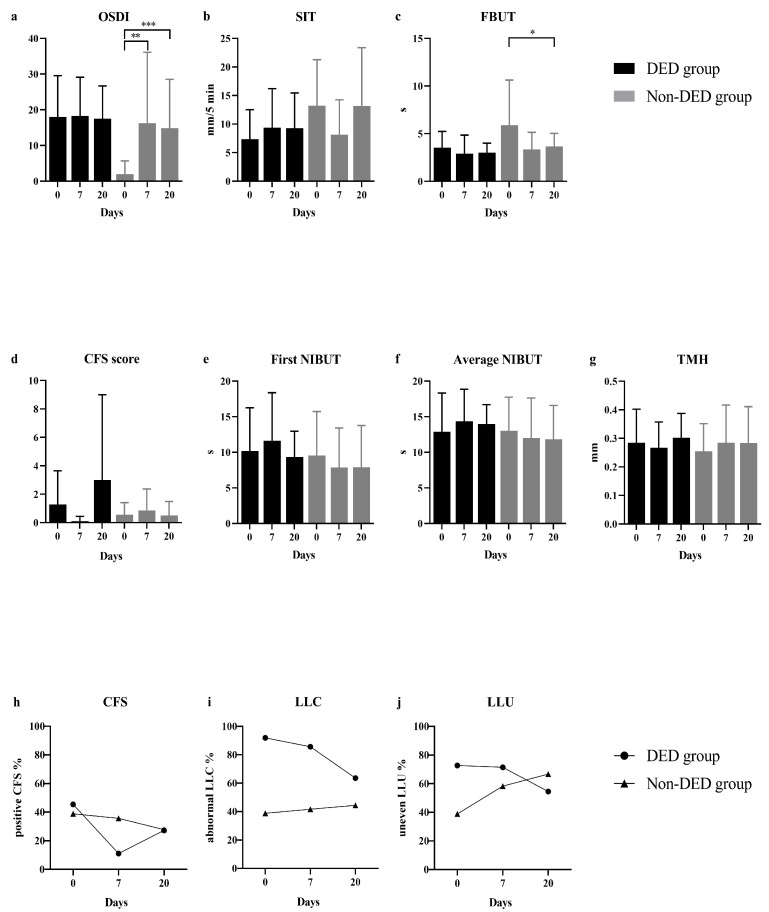
Comparisons of DED parameters before SMILE and on day 7 and day 20 after surgery. OSDI scores (**a**), SIT (**b**) and FBUT (**c**) values in DED patients did not show any significant changing trends before and after surgery. However, an increased OSDI score and significant FBUT reduction were found in non-DED subjects. SIT values in non-DED patients decreased on day 7, and recovered on day 20 without significant difference. The alterations of CFS score (**d**) in both groups did not have any statistically significant differences. The proportion of eyes with positive CFS (**h**) gradually decreased in non-DED group after surgery, while decreased on day 7 and increased on day 20 in DED group. Comparisons on the first NIBUT (**e**), the average NIBUT (**f**) and TMH (**g**) before and after surgery did not find any significant differences in both two groups. Non-DED eyes showed an increasing proportion of abnormal LLC (**i**) and uneven LLU (**j**) after SMILE, which were decreased in DED eyes. * *p* < 0.05, ** *p* < 0.01 and *** *p* < 0.001. DED, dry eye disease; OSDI, ocular surface disease index; SIT, Schirmer I test; FBUT, fluorescein tear film breakup time; CFS, corneal fluorescein staining; NIBUT, non-invasive breakup time; TMH, tear meniscus height; LLC, lipid layer color; LLU, lipid layer uniformity (LLU).

Compared to pre-operative values, a dramatically higher increment of OSDI score (median: 9.09, range: 0.00–66.67, *p* = 0.005) was reported by non-DED subjects on day 7 after SMILE (Figure 1). Non-DED patients also showed a significant FBUT reduction (*p* = 0.048), an increasing proportion of abnormal LLC and uneven LLU on day 20 after surgery. However, such changes were not found in the DED group. The changes of MG loss after surgery were not analyzed because no evidence supported any detectable changes of MG morphology within a short duration in eyes undergoing normal SMILE surgery and routine medical treatments [25].

### 3.3. Regression Analysis

Regression analysis was performed in contact lens wearers because they were more likely to have visual fluctuation due to DED [37]. A total of 14 preoperative parameters were included in the univariate linear regression analysis. It revealed that age and wearing frequency had the highest significance to predict UDVA on 20 days after SMILE (*p* = 0.004 and 0.034, respectively). In addition, wearing duration, daily wearing time, baseline UDVA, CFS score, TMH, and grade score of MG loss potentially influenced UDVA on day 20 after surgery as shown by univariate linear regression analysis (all *p* < 0.35), which were also included in multiple linear regression analysis. It turned out that age, daily wearing time, and preoperative TMH were independent risk factors associated with UDVA in contact lens wearers on day 20 after SMILE (Coef = 0.021, 0.018, and −0.286; *p* = 0.006, 0.010, and 0.043, respectively) (Table 3).

## 4. Discussion

Most patients undergoing refractive surgery used to be contact lens wearers because they had a strong willingness to free themselves from glasses. Moreover, the majority of them had long-term usage of video terminals simultaneously. Nevertheless, both contact lens wearing and overuse of video terminals were the already-known risk factors of DED. It has been confirmed that DED potentially influences the examinations before refractive surgeries and possibly causes measurement biases [9]. Therefore, this study is performed to evaluate the potential of pre-operative DED parameters to predict UDVA and refractive status after SMILE.

Unexpectedly, subjects in DED group were found to have an optometric status closer to emmetropia and greater improvement of UDVA than those in non-DED group on day 20 after SMILE. Although baseline UDVA was worse in DED group, preoperative DED probably affected the outcome in three aspects. First, it is already known that epithelial thinning in DED eyes interferes an accurate preoperative measurement of corneal topography [38]. Moreover, the unstable pre-corneal tear film impairs the optical regularity of corneal epithelium and causes increased ocular forward and corneal backward light scattering [39]. All these factors may affect the accuracy of preoperative optometric examination in the DED group and lead to the potential risk of more cutting thickness than the theoretical value, which causes a refractive status closer to emmetropia on day 20 postoperatively when corneal edema has not completely diminished. Second, DED patients required shorter time to obtain the ocular surface microenvironment recovery to a preoperative level than non-DED patients. Therefore, on day 20 after SMILE, DED eyes were more likely to have a refractive status closer to emmetropia and better UDVA. However, such disparity was temporary and might disappear when the ocular surface is completely recovered in non-DED patients. Third, it should be also noted that patients with preoperative DED usually had better compliance regarding the usage of artificial tears after surgery, which also contribute to a more regular corneal surface and better refractive outcome [40].

Previous studies have revealed that DED is a major ocular surface adverse event leading to dissatisfactory visual recovery after SMILE [24,41,42]. Nevertheless, our study found that patients in the non-DED group had more severely deteriorated DED symptoms (OSDI score) and signs (FBUT) of dry eye after SMILE, which was partly consistent with previous research [43]. Considering the fact that patients with preoperative DED usually have corneal nerve injury due to a worse ocular surface microenvironment and a reduced corneal sensation [10], it was reasonable to deduce that non-DED patients whose corneal innervation and sensation were intact before surgery might have more severe subjective ocular discomfort symptoms, more apparent changes of objective DED signs and larger measurement bias due to newly developed DED after SMILE than those who already had DED before surgery.

Our study revealed that older age, longer contact lens daily wearing time and lower TMH were the risk factors associated with worse prediction of UDVA after SMILE in contact lens wearers. Since the incidence of DED increases with age [12,13], it is reasonable to suppose that older subjects have a worse ocular surface microenvironment, and need a longer time to have physiological homeostasis of tear film restored. It has been proven that the component of tear film (including lipid layer, aqueous layer and mucin layer) as well as tear dynamics were both affected in long-term contact lens wearers [44,45,46,47,48] and its degree positively correlated with the length of contact lens wearing time [49]. Containing 75%~90% of total tear volume [50,51], TMH is considered as a sensitive indicator of tear fluid volume and used in the diagnosis of aqueous deficient DED [52,53]. Our previous study showed that wearing contact lenses caused a reduction of TMH and decreased area of lower tear meniscus [54], and lower TMH correlated with shorter NIBUT [55]. Therefore, lower TMH potentially affects the accuracy of preoperative measurement and had a negative impact on visual recovery after SMILE [56,57].

Several limitations should be acknowledged. First, the sample size of our study is not large enough, which likely contributes to the lack of statistical significance for some parameters. Second, the follow-up is not long enough to evaluate the long-term impact of pre-operative DED on UDVA and visual recovery after SMILE. Further multicenter studies with a larger sample size and longer follow-up are needed.

## 5. Conclusions

In conclusion, age, contact lens daily wearing time, and preoperative TMH are the three dependent factors to predict LogMAR UDVA on day 20 after SMILE in contact lens wearers. Pre-operative existing DED and SMILE affect each other. The current study provides supporting evidence that the impact of DED on the prediction of UDVA and refraction should be taken into consideration by refractive surgeons before SMILE so as to make individualized therapies for patients with pre-operative DED.

## Figures and Tables

**Table 1:** Preoperative demographic data and clinical characteristics of DED and non-DED group.

	DED Group (*n* = 11)	Non-DED Group (*n* = 18)	*p* Value
Age (yrs)	23.27 ± 2.76	26.50 ± 6.35	0.071
Gender (male/female)	4/7	8/11	0.717
Eye (OD/OS)	6/5	9/9	1.000
Wearing CL (SCL/RGP/non)	6/0/5	8/1/9	1.000
Wearing duration (yrs)	4.92 ± 0.97	5.50 ± 4.17	0.696
Wearing frequency (days/wk)	0.23 (0.03–5.00)	2.60 ± 2.83	0.262
Daily wearing time (hrs)	8.67 ± 1.75	7.72 ± 2.69	0.464
LogMAR UDVA	1.19 ± 0.15	0.98 ± 0.36	0.036
SE (D)	−5.56 ± 1.37	−4.78 ± 1.76	0.225
Dry eye related parameters			
OSDI	14.58 (5.56–40.00)	1.94 ± 3.74	<0.001
SIT (mm/5 min)	7.36 ± 5.16	13.22 ± 8.06	0.041
FBUT (s)	3.55 ± 1.69	4.00 (2.00–18.00)	0.162
Positive CFS	45.45% (5/11)	38.89% (7/18)	1.000
CFS score	0.00 (0.00–8.00)	0.00 (0.00–3.00)	0.543
First NIBUT (s)	10.16 ± 6.09	7.27 (2.29–20.20)	0.574
Average NIBUT (s)	12.90 ± 5.42	13.01 ± 4.74	0.957
TMH (mm)	0.25 (0.15–0.52)	0.25 ± 0.10	0.404
Grade score of MG loss	2.00 (1.00–6.00)	1.67 ± 0.91	0.365
Abnormal LLC	90.91% (10/11)	38.89% (7/18)	0.008
Uneven LLU	72.73% (8/11)	38.89% (7/18)	0.128

DED, dry eye disease; CL, contact lens; SCL, soft contact lens; RGP, rigid gas permeable contact lens; UDVA, uncorrected distance visual acuity; SE, spherical equivalent; OSDI, ocular surface disease index; SIT, Schirmer I test; FBUT, fluorescein tear film breakup time; CFS, corneal fluorescein staining; NIBUT, non-invasive breakup time; TMH, tear meniscus height; MG, meibomian gland; LLC, lipid layer color; LLU, lipid layer uniformity (LLU).

**Table 2:** Comparison of UDVA, SE and DED parameters between DED and Non-DED group on day 20 after surgery.

	DED Group (*n* = 11)			Non-DED Group (*n* = 18)			*p* Value (DED vs. Non-DED on Day 20)	*p* Value (DED vs. Non-DED: Pre- Postoperative Alterations)
	Day 20 after Surgery	Pre-Postoperative Alterations	*p* Value (Pre- vs. Day 20)	Day 20 after Surgery	Pre-Postoperative Alterations	*p* Value (Pre- vs. Day 20)		
LogMAR UDVA	−0.05 ± 0.07	−1.24 ± 0.18	<0.001	−0.01 ± 0.09	−1.00 (−2.00–(−0.48))	<0.001	0.248	0.008
SE (D)	−0.01 ± 0.39	5.55 ± 1.23	<0.001	−0.34 ± 0.35	4.44 ± 1.80	<0.001	0.026	0.085
OSDI	17.49 ± 9.20	−0.52 ± 15.00	0.911	9.76 (0.00–56.82)	8.30 (0.00–52.65)	<0.001	0.333	0.031
SIT (mm/5 min)	9.27 ± 6.17	1.91 ± 7.01	0.387	13.17 ± 10.21	−0.06 ± 7.49	0.975	0.265	0.489
FBUT (s)	3.00 ± 1.00	−0.55 ± 1.57	0.277	3.00 (2.00–7.00)	−1.00 (−11.00–2.00)	0.048	0.183	0.424
Positive CFS	27.27% (3/11)	−18.18% (−2/11)	0.625	27.78% (5/18)	−11.11% (−2/18)	0.754	1.000	1.000
CFS score	0.00 (0.00–15.00)	−1.00 (−8.00–15.00)	0.814	0.00 (0.00–3.00)	−0.06 ± 1.39	0.868	0.732	0.743
First NIBUT (s)	9.33 ± 3.64	−0.84 ± 6.95	0.698	5.26 (2.48–23.00)	−1.63 ± 6.95	0.333	0.200	0.767
Average NIBUT (s)	13.97 ± 2.72	1.07 ± 4.31	0.428	11.83 ± 4.77	−1.18 ± 6.29	0.438	0.135	0.306
TMH (mm)	0.30 ± 0.09	0.02 ± 0.14	0.688	0.28 (0.14–0.67)	0.00 (−0.12–0.43)	0.727	0.345	0.822
Abnormal LLC	63.64% (7/11)	−27.27% (−3/11)	0.250	44.44% (8/18)	5.56% (1/18)	1.000	0.450	0.693
Uneven LLU	54.55% (6/11)	−18.18% (−2/11)	0.625	66.67% (12/18)	27.78% (5/18)	0.180	0.696	0.197

UDVA, uncorrected distance visual acuity; SE, spherical equivalent; DED, dry eye disease; Pre-Postoperative alterations, the alterations between preoperative baseline and postoperative value; OSDI, ocular surface disease index; SIT, Schirmer I test; FBUT, fluorescein tear film breakup time; CFS, corneal fluorescein staining; NIBUT, non-invasive breakup time; TMH, tear meniscus height; LLC, lipid layer color; LLU, lipid layer uniformity (LLU).

**Table 3:** Linear regression to determine preoperative predictors of UDVA on day 20 after SMILE in contact lens wearers.

		R^2^/Coef	*p* Value
Univariate linear regression	Age	0.493	0.004
	Wearing duration	0.094	0.266
	Wearing frequency	0.302	0.034
	Daily wearing time	0.138	0.172
	LogMAR UDVA	0.186	0.108
	SE	0.009	0.744
	OSDI	0.052	0.415
	SIT	0.037	0.494
	FBUT	0.010	0.727
	CFS score	0.111	0.225
	First NIBUT	0.028	0.549
	Average NIBUT	0.038	0.485
	TMH	0.072	0.334
	Grade score of MG loss	0.074	0.326
Multiple linear regression	Age	0.021	0.006
	Wearing duration	−0.012	0.053
	Wearing frequency	−0.009	0.097
	Daily wearing time	0.018	0.010
	LogMAR UDVA	−0.002	0.982
	CFS score	−0.004	0.564
	TMH	−0.286	0.043
	Grade score of MG loss	−0.002	0.917
	Cons	−0.540	0.029

UDVA, uncorrected distance visual acuity; SMILE, small incision lenticule extraction; SE, spherical equivalent; OSDI, ocular surface disease index; SIT, Schirmer I test; FBUT, fluorescein tear film breakup time; CFS, corneal fluorescein staining; NIBUT, non-invasive breakup time; TMH, tear meniscus height; MG, meibomian gland; LLU, lipid layer uniformity (LLU); Cons, constant.

## Data Availability

All data are contained within the article.
